# Metagenomic Sequencing and Reverse Transcriptase PCR Reveal That Mobile Phones and Environmental Surfaces Are Reservoirs of Multidrug-Resistant Superbugs and SARS-CoV-2

**DOI:** 10.3389/fcimb.2022.806077

**Published:** 2022-03-08

**Authors:** Syrine Boucherabine, Rania Nassar, Shroque Zaher, Lobna Mohamed, Matthew Olsen, Fatma Alqutami, Mahmood Hachim, Abdulmajeed Alkhaja, Mariana Campos, Peter Jones, Simon McKirdy, Rashed Alghafri, Lotti Tajouri, Abiola Senok

**Affiliations:** ^1^ College of Medicine, Mohammed Bin Rashid University of Medicine and Health Sciences, Dubai, United Arab Emirates; ^2^ Oral and Biomedical Sciences, School of Dentistry, College of Biomedical and Life Sciences, Cardiff University, Cardiff, United Kingdom; ^3^ Faculty of Health Sciences and Medicine, Bond University, Robina, QLD, Australia; ^4^ Medical Education & Research Department, Dubai Health Authority, Dubai, United Arab Emirates; ^5^ CSIRO Land and Water, CSIRO Health and Biosecurity, Floreat, WA, Australia; ^6^ Harry Butler Institute, Murdoch University, Murdoch, WA, Australia; ^7^ General Department of Forensic Sciences and Criminology, Dubai Police, Dubai, United Arab Emirates; ^8^ Dubai Future Council on Community Security and Dubai Police Scientists Council, Duba, United Arab Emirates

**Keywords:** mobile phones, fomites, shotgun metagenomics, SARS-CoV-2, *Pseudomonas*, *Staphylococcus*

## Abstract

**Background:**

Mobile phones of healthcare workers (HCWs) can act as fomites in the dissemination of microbes. This study was carried out to investigate microbial contamination of mobile phones of HCWs and environmental samples from the hospital unit using a combination of phenotypic and molecular methods.

**Methods:**

This point prevalence survey was carried out at the Emergency unit of a tertiary care facility. The emergency unit has two zones, a general zone for non-COVID-19 patients and a dedicated COVID-19 zone for confirmed or suspected COVID-19 patients. Swabs were obtained from the mobile phones of HCWs in both zones for bacterial culture and shotgun metagenomic analysis. Metagenomic sequencing of pooled environmental swabs was conducted. RT-PCR for SARS-CoV-2 detection was carried out.

**Results:**

Bacteria contamination on culture was detected from 33 (94.2%) mobile phones with a preponderance of *Staphylococcus epidermidis* (*n*/*N* = 18/35), *Staphylococcus hominis* (*n*/*N* = 13/35), and *Staphylococcus haemolyticus* (*n*/*N* = 7/35). Two methicillin-sensitive and three methicillin-resistant *Staphylococcus aureus*, and one pan-drug-resistant carbapenemase producer *Acinetobacter baumannii* were detected. Shotgun metagenomic analysis showed high signature of *Pseudomonas aeruginosa* in mobile phone and environmental samples with preponderance of *P. aeruginosa* bacteriophages. *Malassezia* and *Aspergillus* spp. were the predominant fungi detected. Fourteen mobile phones and one environmental sample harbored protists. *P. aeruginosa* antimicrobial resistance genes mostly encoding for efflux pump systems were detected. The *P. aeruginosa* virulent factor genes detected were related to motility, adherence, aggregation, and biofilms. One mobile phone from the COVID-19 zone (*n*/*N* = 1/5; 20%) had positive SARS-CoV-2 detection while all other phone and environmental samples were negative.

**Conclusion:**

The findings demonstrate that mobile phones of HCWs are fomites for potentially pathogenic and highly drug-resistant microbes. The presence of these microbes on the mobile phones and hospital environmental surfaces is a concern as it poses a risk of pathogen transfer to patients and dissemination into the community.

## Introduction

Nosocomial infections constitute an important health problem with high incidence, morbidity, and mortality rates worldwide (Ak et al., 2011). Multidrug-resistant (MDR) bacteria are one of the most important threats to public health and are typically associated with nosocomial infections (van Duin and Paterson, 2016). Despite their best intentions, healthcare workers (HCWs) may inadvertently act as vectors, disseminating infectious agents including MDR microbes within the healthcare setting. As HCWs are a bridge between the hospital and the community with daily interaction within both settings, they may also carry a multitude of pathogens from the hospital into the community. Environmental screening has shown that contamination of surfaces and items still occurs in clinical care areas in healthcare settings despite cleaning protocols (Lemmen et al., 2004; Saka et al., 2019). HCWs are liable to touch these contaminated surfaces during patient care, which increases the risk of onward transmission to others (Bhalla et al., 2004; Lemmen et al., 2004).

In the highly digital and technologically interconnected age we live in, one of the often-ignored high touch surfaces is the personal digital devices of HCWs. In a recent report from Italy, 93% of health profession students used their mobile phones on the wards, whereas only 11% changed gloves between medical procedures and the use of phone with just 3% routinely cleaning their phones (D’Alo et al., 2020). Similarly, in a study by Cavari et al. (2016), 90% of participating HCWs kept their phones with them in the workplace with up to 37% using their mobile phones at least once every hour during their shift and only 13% reported regular disinfection of their devices. In a recent report of a survey of healthcare workers in an acute pediatric healthcare setting in Australia, 56% (86/165) of respondents indicated that they use their phones in the bathroom, which demonstrates the use of these devices in unhygienic environments (Olsen et al., 2021a). These habits are of significant concern as they potentially reduce or nullify the effect of hand hygiene practices in limiting the spread of microbes in the healthcare setting. Indeed, mobile phones have been identified as “Trojan horses” in propagating pathogens, including viruses especially during outbreaks and pandemics (Pillet et al., 2016; Amanah et al., 2019; Olsen et al., 2020). Previous studies on mobile phone contamination have used conventional culture-based methods and reported a preponderance of Gram-positive bacteria in particular staphylococcal species (Selim and Abaza, 2015; Jansen et al., 2019; Khashei et al., 2019; Hikmah and Anuar, 2020; Olsen et al., 2020; Qureshi et al., 2020). However, findings from a recent study using a 16s rRNA sequencing approach and shotgun metagenomics indicates that mobile phones of HCWs are contaminated with a higher diversity of bacteria than previously reported (Simmonds et al., 2020; Olsen et al., 2021b; Tajouri et al., 2021) with greater abundance of Gram-negative organisms (Simmonds et al., 2020; Tajouri et al., 2021).

With the COVID-19 pandemic, a shift towards higher usage of digital devices including mobile phones for accessing and documenting information in the healthcare setting has been encouraged. This could potentially increase the risk of occurrence of contamination with and transmission of microbial agents including SARS-CoV-2. Indeed, survival of SARS-CoV-2 for up to 28 days on vinyl and glass, which are materials commonly associated with mobile phones, has been demonstrated (Riddell et al., 2020). However, the impact of the heightened pandemic related hand hygiene and environmental disinfection measures in mitigating against this risk remains unknown. Therefore, in the context of the ongoing pandemic, this study was carried out to investigate the microbial contamination of mobile phones of HCWs using a combination of phenotypic, molecular, and shotgun next-generation sequencing metagenomic approaches. In addition, mobile phone utilization and disinfection by HCWs during their work shift was also assessed.

## Methods

### Study Site

The study was carried out at the Emergency care unit, Rashid Hospital, Dubai, and Mohammed Bin Rashid University of Medicine and Health Sciences (MBRU), Dubai Healthcare City in the United Arab Emirates (UAE). Rashid Hospital is a 771-bed tertiary public healthcare facility under the auspices of Dubai Health Authority. The emergency unit has two zones, a general zone for non-COVID-19 patients and a dedicated COVID-19 zone for confirmed or suspected COVID-19 patients. Ethical approval for the study was granted by the Dubai Scientific Research Ethics Committee, Dubai Health Authority (REF#: DSREC-02/2021_02) and MBRU Institutional Review Board (REF#: MBRU-IRB-2020-040). In accordance with the ethical approval, the study was explained to participants and verbal consent was obtained. Prior to collection of samples, participants were required to indicate using a check box on the questionnaire that they had given verbal consent.

### Specimen Collection

The study was designed as a point prevalence survey hence the Emergency unit, Rashid Hospital was visited once during the busy morning shift and all HCWs on duty in both zones at the time of the visit participated. The HCWs did not have any prior knowledge of the research team’s visit for sample collection.

*Mobile phones:* Mobile phones of the HCWs were sampled and a questionnaire to obtain demographic profile, usage, and cleaning of mobile phones during the work shift was administered. The mobile phones sampled were flat screened smartphones without keypads. Gloves were worn when handling and swabbing the mobile phones and replaced after each swab sample to prevent cross-contamination. The swabs were pre-moistened with sterile normal saline and swabbing was carried out with care to ensure that the applicator tip made contact with the front and back of the devices. Two swabs were obtained per device with the first sample (which was used for Next-generation sequencing and SARS-CoV-2 detection) placed in Zymos DNA/RNA Shield Collection Tubes (Zymos Research, Irvine CA, USA) and the second swab (used for bacterial culture) was placed in Culture Swab EZ IITM” (Becton Dickinson) tubes.

*Environmental samples:* To get a snapshot of the microbial contamination in the emergency unit, environmental surfaces in the COVID-19 and General zones were swabbed and pooled for shotgun metagenomics and for SARS-CoV-2 detection. The surfaces swabbed were desks, computer keyboards, landline phones in the nursing station, treatment room, consulting rooms, as well as patient bed railings. Pools 1 and 2 are from the COVID-19 zone while Pools 3 and 4 are from the general zone. Each pooled sample consisted of swabs obtained from five different surfaces in the respective zone. All collected swabs (environmental and mobile phones) were sent to the MBRU Microbiology Research Laboratory for processing.

### Bacterial Culture

The swabs from the Culture Swab EZ IITM tubes were incubated in brain heart infusion broth (BHI) for 24–48 h, followed by culture on blood agar, mannitol salt agar, and McConkey agar plates. Gram stain and biochemical tests of characteristic colonies were carried out and followed by subculture to obtain pure colonies. Confirmation of bacterial identification and antibiotic susceptibility profile was carried out using the Vitek automated method (BioMerieux Marcy L’Etoile France).

### DNA Microarray

Molecular characterization of *Staphylococcus aureus* isolates was carried out using the StaphyType DNA microarray (Alere Technologies GmbH, Jena, Germany). Briefly, *S. aureus* isolates sub-cultured on Columbia blood agar were harvested and DNA extraction was carried out using Qiagen DNA extraction kits (Qiagen, Hilden, Germany). The DNA microarray was carried out using target genes, primer, and probe sequences in accordance with manufacturer-provided protocol as previously reported (Monecke et al., 2011a; Monecke et al., 2011b; Monecke et al., 2016; Senok et al., 2016). Microarray images were taken and analyzed using the dedicated reader and software (Alere Technologies, Germany). Assignment to clonal complexes, sequence types, and identification of epidemic strains were carried out as previously described (Senok et al., 2016).

### Shotgun Metagenomics

This was carried out at CosmosID Laboratories (Rockville, MD). Briefly, DNA extraction was carried out using the QIAmp^®^ Powersoil Pro kit (Qiagen, Hilden, Germany) and isolated DNA was quantified by Qubit (ThermoFisher, Waltham MA, USA). The DNA libraries were prepared using the Nextera XT DNA Library Preparation Kit (Illumina, San Diego CA, USA) and IDT Unique Dual Indexes with total DNA input of 1 ng. Genomic DNA was fragmented using a proportional amount of Illumina Nextera XT fragmentation enzyme. Combinatory dual indexes were added to each sample followed by 12 cycles of PCR to construct libraries. DNA libraries were purified using AMpure magnetic Beads (Beckman Coulter, Brea CA, USA) and eluted in QIAGEN EB buffer. Library quantity was assessed with Qubit (ThermoFisher). Libraries were then sequenced on an Illumina HiSeq 4000 platform 2×150bp (Illumina). Unassembled sequencing reads were directly analyzed by CosmosID bioinformatics platform for multi-kingdom microbiome analysis and profiling of antibiotic resistance and virulence genes and quantification of organisms’ relative abundance (Olsen et al., 2021b). Each sample output of sequencing raw data of millions of short reads were distinctively aligned against sequences of microbial genomes for high level of taxonomic resolution identification. Microbial “Richness” was analyzed by determining the cumulative amount of any single distinct microbial species or strain within a particular set of samples (e.g., mobile phone cohort alone, or the environment cohort alone, or both cohorts together). The richness was reported as Hits, which correspond to the number of occurrences of each distinct microbe (strain or species) retrieved across a group of samples. Metagenomics Sequence data are available on NCBI (https://www.ncbi.nlm.nih.gov/genbank/ BioProject ID: PRJNA750471).

### SARS-CoV-2 Detection

Viral RNA extraction was carried out using Qiagen QIAamp Viral Mini kit followed by SARS-CoV-2 Reverse Transcriptase PCR using the PerkinElmer New Coronavirus Nucleic Acid Detection Kit (Waltham MA, USA) in accordance with manufacturer-provided protocol.

## Results

### Participants’ Demographics, Mobile Phone Utilization, and Disinfection Practices

Swab samples were obtained from mobile phones of 35 HCWs in the emergency care unit (30 from the general zone and 5 from the COVID-19 zone). All the HCWs were part of the morning shift except one who was ending her shift. The majority of HCWs use their mobile phones while at work and consider it essential for their work (**Table 1**). The demographic data of participants as well as their mobile phone utilization and cleaning habits are shown in **Table 1**.

**Table 1:** Demographic profile and the mobile phone utilization and cleaning practices of participants.

Total number of participants		*N* = 35
Age range	18–25	5 (14.3%)
	26–55	28 (80.0%)
	>55 years	2 (5.7%)
Gender	Male	14 (40.0%)
	Female	13 (37.1%)
	Not indicated	8 (22.9%)
Staff category	Nurse	21 (60.0%)
	Physician	8 (22.9%)
	Others	6 (17.1%)
Use of mobile phone during the work shift	Yes	29 (82.9%)
	No	6 (17.1%)
Mobile phone considered essential for work purposes	Yes	28(80.0%)
	No	7 (20.0%)
Last time mobile phone was cleaned		
	Within the last 1 h	10 (28.6%)
	>1 h but within last 24 h	17 (48.5%)
	>24 h but within the last 1 week	7(20.0%)
	> One week	1 (2.9%)
What was used for cleaning the mobile phones	Alcohol swab/disinfectant spray	27 (77.1%)
	Dry cloth/non-disinfectant wipe	8 (22.9%)
Use of mobile phone in the toilet	Yes	21 (60.0%)
	No	14 (40.0%)
Mobile phones can harbor microbes	Yes	29 (82.8%)
	No	1 (2.9%)
	Unsure	4 (11.4%)
	No response	1 (2.9%)

### Bacterial Culture and DNA Microarray

All mobile phone swabs were processed for bacterial culture. Bacterial contamination was detected in 94.2% (*n* = 33) of mobile phones swabbed. Among the contaminated mobile phones, only one microorganism was cultured from 30.3% (*n* = 10), while majority (60.6%; *n* = 20) harbored two microorganisms. The predominant organisms identified from culture were *Staphylococcus epidermidis* detected in 51.4% (*n*/*N* = 18/35), *Staphylococcus hominis* in 37.1% (*n*/*N* = 13/35), and *Staphylococcus haemolyticus* in 20% (*n*/*N* = 7/35) of devices sampled (**Table 2**). Multidrug-resistant and opportunistic pathogens identified from the mobile phones include methicillin-resistant *S. aureus* (MRSA), *Acinetobacter baumannii, Pseudomonas luteola*, and *Panotoea* spp. (**Table 2**). Of the two *A. baumannii* isolates identified, one was a pan-drug-resistant carbapenemase producer with resistance to piperacillin, piperacillin-taxobactam, ceftazidime, cefepime, azetronam, imipenem, meropenem, and intermediate resistance to ticarcillin and ticarcillin-clavulanic acid. Methicillin resistance phenotype was detected in 44.4% (*n*/*N* = 8/18) of the *S. epidermidis* isolates.

**Table 2:** Distribution of bacteria isolated from mobile phones and identified by culture-based methods.

Bacteria	Number of contaminated devices
Staphylococcus epidermidis*	20
*Staphylococcus hominis*	13
*Staphylococcus haemolyticus*	7
*Micrococcus luteus*	2
Methicillin-resistant *Staphylococcus aureus*	3
Methicillin-sensitive *Staphylococcus aureus*	2
*Staphylococcus warneri*	2
*Staphylococcus capitis*	2
*Streptococcus mitis/oralis*	2
*Acinetobacter baumannii*	2
*Streptococcus sanguinis; Aerococcus viridans; Kocuria kristinae; Staphylococcus lugdunensis; Pseudomonas luteola; Panotoea* spp.	1**

*8 isolates had a methicillin-resistant phenotype characterized by cefoxitin positive and oxacillin resistance.

**Each microorganism was detected on a single mobile phone.

DNA microarray was performed on the 5 *S. aureus* isolates detected. DNA microarray analysis identified the methicillin-sensitive *S. aureus* (MSSA) isolates as CC15-MSSA and CC361-MSSA. All the three MRSA were CC1 strains, namely, CC1-MRSA-[V+*fusC*+*ccrAB1*] (*n* = 2) and CC1-MRSA-[V/VT+*fusC*+*ccrAB1*] (*n* = 1). Details of the molecular characterization of the *S. aureus* strains is shown in **Supplementary Table 1**.

### Microbial Diversity From Shotgun Metagenomics Analysis

The average read sequencing across all 39 metagenomics 35 for the mobile phone cohort and 4 for the environmental cohort was 47 million reads (highest: 116 million and lowest: 7.9 million sequencing reads). The average read sequencing output for the mobile phone cohort and the environmental cohort was 61 and 78 million sequencing reads, respectively.

**Figure 1:**
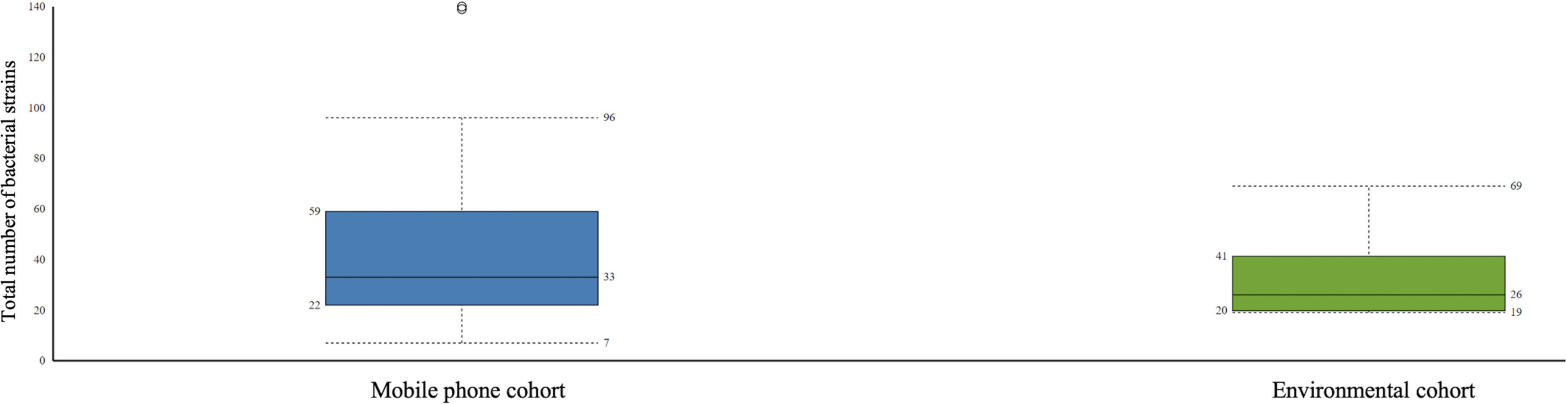
Boxplot representation of the statistics of bacteria strains found in the mobile phone cohort versus the environmental cohort. Interquartile ratio values of 37 and 21 defined the number of bacteria strains in mobile phone cohort and the environmental cohort, respectively.

*Bacteria:* Bacteria species detected were 358 in the mobile phone cohort and 81 in the environmental cohort. A total of 609 different bacteria strains were found across the combined mobile phone cohort and environmental cohort, namely, 517 in the mobile phone cohort versus 92 in the environmental cohort. These three cohorts, namely, combined mobile phone and environmental cohorts, mobile phone cohort only, and environmental cohort only, accounted for 1,668, 1,528, and 140 hits, which represent an average of 42.8, 43.7, and 8.8 individual bacteria per sample, respectively. The strains with the highest hits score are shown in **Table 3**. **Figure 1** illustrates a box plot representation of the bacterial strains identified on mobile phones and the environment. The median values of bacteria strains found in the mobile phone cohort was 33 versus 36 in the environmental cohort (**Figure 1**). By the means of a principal component analysis of the mobile phone cohort (*n* = 35), 3 outliers were found apart from a dense cluster of the remaining other 32 mobile phones (**Supplementary Figure**). Analysis of these three mobile phone outliers showed that all 3 phones consistently harbor a total of 31 different strains (100%, 3/3) (**Supplementary Table 2**).

**Table 3:** Richness of top individual bacterial strain hits found in the mobile phone and environmental cohorts on metagenomic sequencing.

Mobile phone cohort (*N* = 35)		Environmental cohort (*N* = 4)	
Hits % (*n*/*N*)	Organism	Hits % (*n*/*N*)	Organism
100% (35/35)	*Pseudomonas aeruginosa 6077*	100% (4/4)	*Pseudomonas aeruginosa 6077*
100% (35/35)	*Pseudomonas* sp. *HMSC063H08*	100% (4/4)	*Pseudomonas* sp. *HMSC063H08*
97% (34/35)	*Aquabacterium parvum*	100% (4/4)	*Aquabacterium parvum*
97% (34/35)	*Pseudomonas_u_t*	100% (4/4)	*Pseudomonas_u_t*
94% (33/35)	*Agrobacterium tumefaciens F2*	100% (4/4)	*Agrobacterium tumefaciens F2*
74% (26/35)	*Micrococcus luteus*	100% (4/4)	*Micrococcus luteus*
74% (26/35)	*Micrococcus aloeverae*	100% (4/4)	*Paracoccus aeridis*
71% (25/35)	*Micrococcus yunnanensis*	75% (3/4)	*Micrococcus aloeverae*
66% (23/35)	*Propionibacteriaceae_u_t*	75% (3/4)	*Propionibacterium* sp. *HMSC065F07*
63% (22/35)	*Stenotrophomonas* sp. *MB339*	75% (3/4)	*Halomonas desiderata SP1*

**Figure 2:**
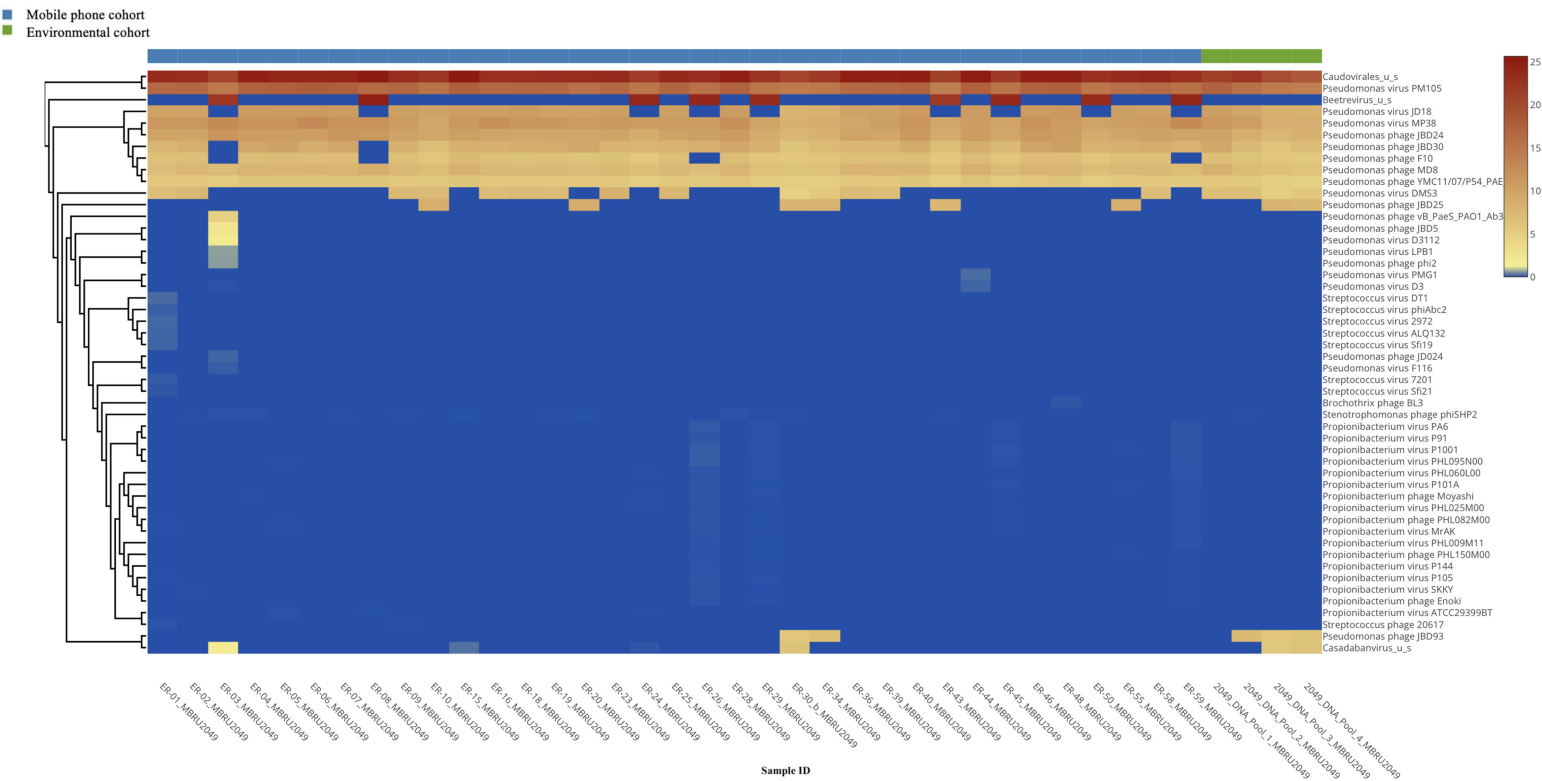
Heatmap of the bacteriophage distribution.

**Figure 3:**
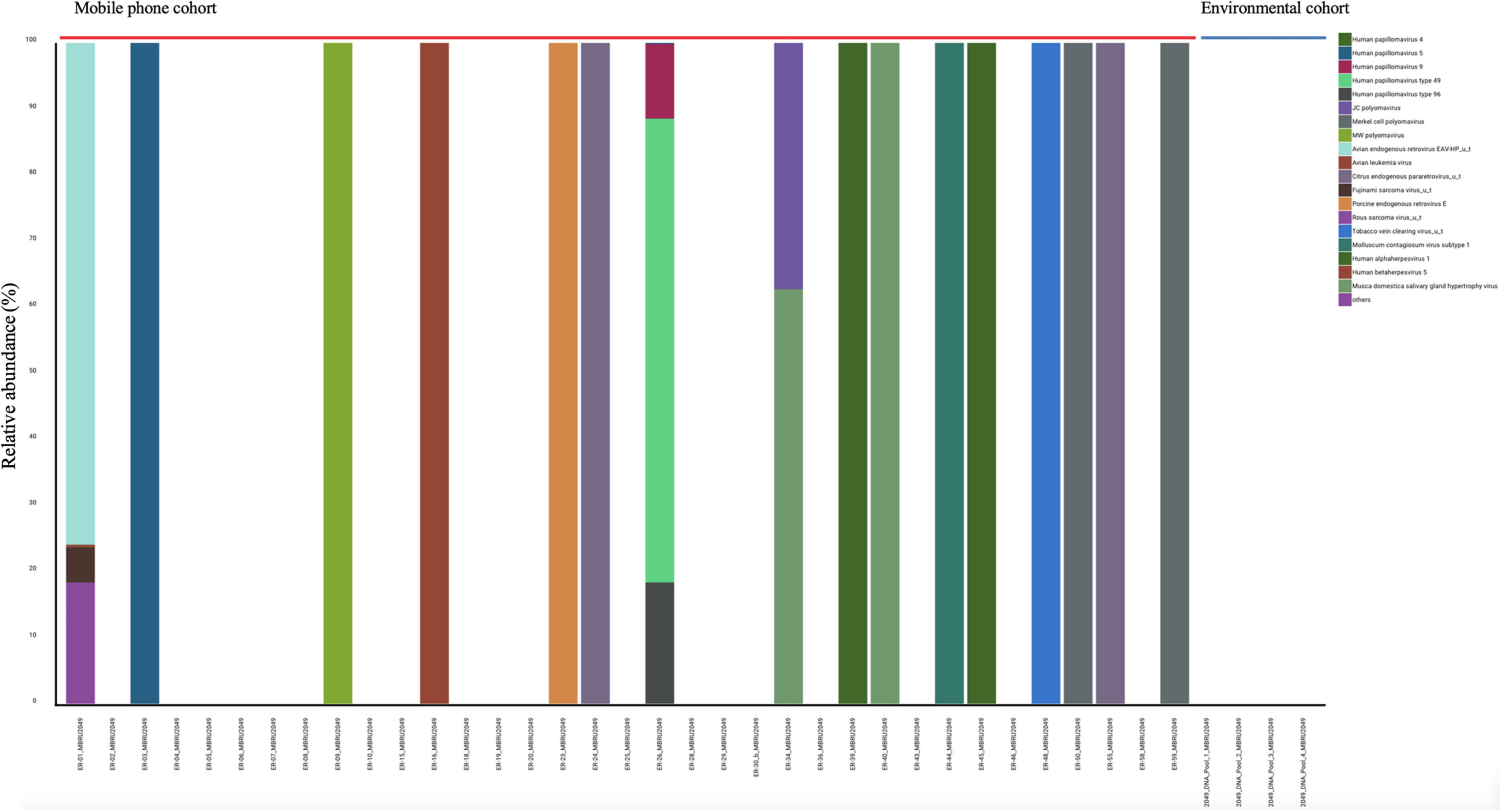
Stacked bar representation of types and relative abundances of viruses found by metagenomic analysis. Mobile phone cohort (*N* = 35) and the Environmental cohort (*N* = 4).

*Viruses:* All 35 mobile phone swabs and all four environmental swabs harbored bacteriophages (**Figure 2**) with high predominance of *P. aeruginosa* bacteriophages. Apart from bacteriophages, other viruses were found in 19/35 (54.2%) of mobile phone swabs whereas none were found in the four environmental samples (**Figure 3**).

*Fungi:* A total of 45 and 12 fungal species were found in the mobile phone cohort (*n* = 35) and environmental cohort (*n* = 4), respectively. The presence of these different fungi accounted for 198 (an average of 5.7 per mobile phone) and 22 (an average of 5.5 per environmental swab) found in the mobile phone cohort and environmental cohort respectively. The predominant fungi found in mobile phone and environmental samples were *Malassezia* species (mobile phone: *n*/*N* = 32/35; environmental: *n*/*N* = 4/4) and *Aspergillus* species (mobile phone: *n*/*N* = 9/35; environmental: *n*/*N* = 2/4). *Candida parapsilosis* was found only in mobile phone samples (*n*/*N* = 4/35; 11%).

*Protists:* A total of 14 mobile phones had protists with 12 phones having one protist and two phones harboring two strains. Only three strains could be identified with the most prevalent on phones being *Pseudoperonospora cubensis* (richness of 10 hits) followed by *Acanthamoeba polyphaga* (on 5 mobile phones) and *Acanthamoeba mauritaniensis*, which was identified in only one phone. Only one environmental swab harbored a protist, which was *Pseudoperonospora cubensis*.

**Figure 4:**
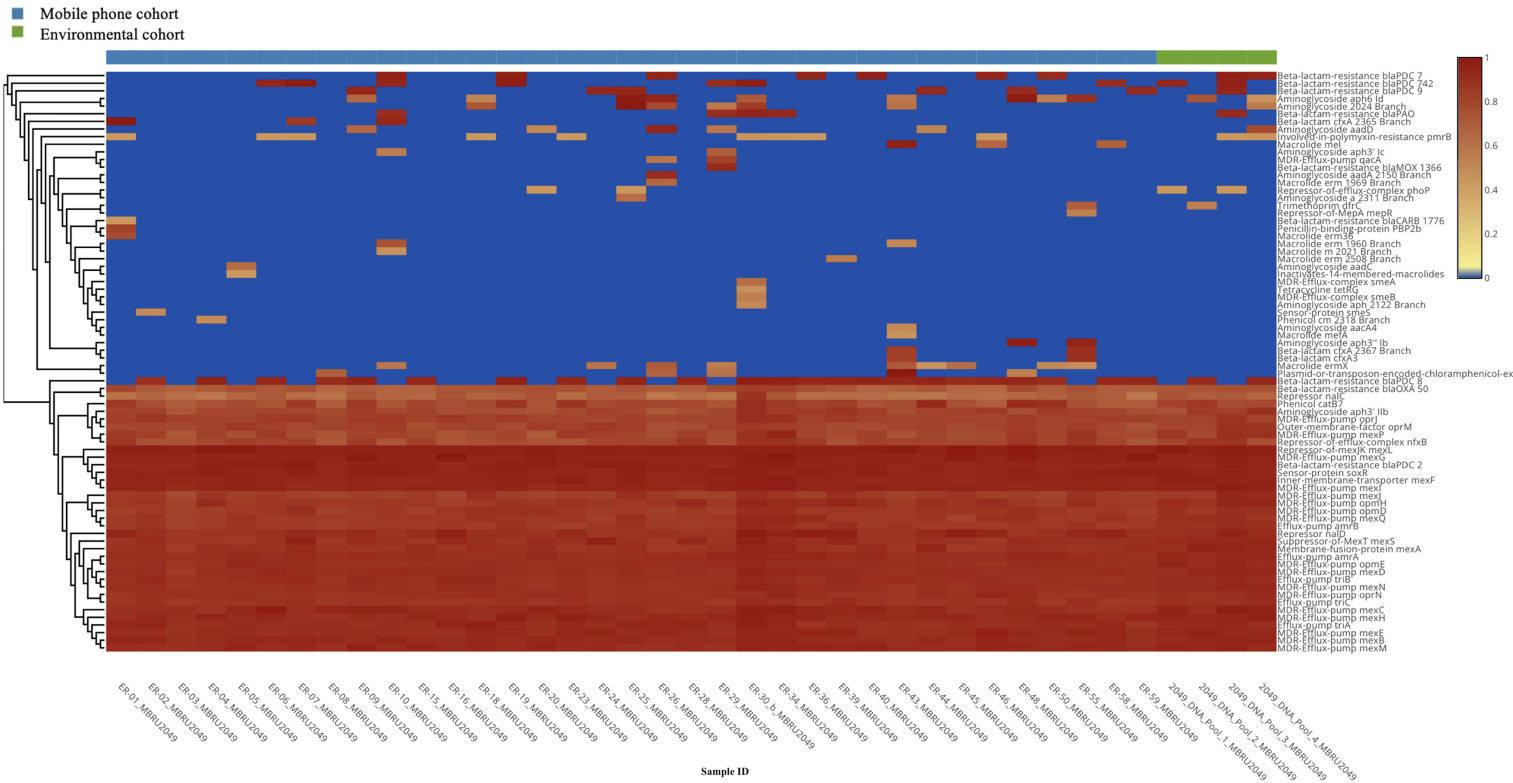
Heatmap representation of the antibiotic resistance genes (ARGs) identified by metagenomic analysis of both mobile phone cohort (*n* = 35 mobile phone swabs) and environmental cohort (*n* = 4 environmental swabs, in green).

**Figure 5:**
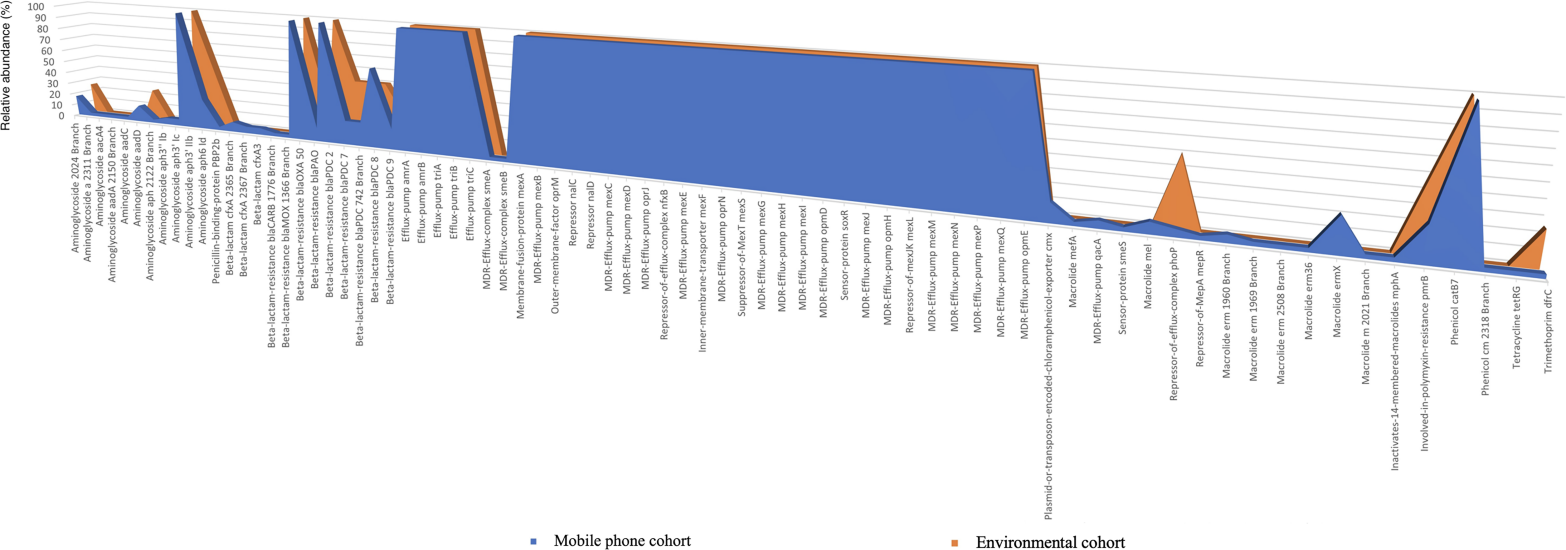
Antibiotic resistance genes (ARGs) present on the mobile phone cohort (*n* = 35) and the environmental cohort (*n* = 4). The *x*-axis shows the type of antibiotic resistance gene identified in the metagenomic analysis. The *y*-axis is the percentage of occurrences of such gene relative to the cohorts’ sample number. 100% indicates that a particular gene is present in all the mobile phone cohort (*n* = 35 mobile phone swabs) in blue or in all environmental cohort (*n* = 4 environmental swabs) in orange.

*Antibiotic resistance genes (ARGs):* A total of 76 (richness of 1,356 hits; average = 38.74 genes per mobile phone) and 46 (richness of 157 hits; average = 39.25 genes per environmental swab) different ARGs were found in the mobile phone cohort and environmental cohort, respectively. A predominant presence of *P. aeruginosa* ARGs was found (**Figure 4**). All 39 mobile phones and environmental swab samples harbored the common presence of a total of 35 consistent genes In the environmental cohort, 11 ARGs are not consistently present on all 4 environmental swabs. **Figure 5** compares the relative abundance of the ARGs identified across all 35 mobile phones and the 5 environmental samples. In the mobile phone cohort, 41 ARGs were not consistently present on all mobile phone swabs (**Figure 5**). Metagenomic analysis of the mobile phone cohort and the environmental cohort revealed genes encoding for multidrug (MDR) efflux pump systems. Genes encoding for resistance-nodulation-cell division type (RND) tripartite efflux pumps detected in all the mobile phone and environmental swabs were *amrAB-oprM*, *abc-opmH*, *mexAB-oprM*, *mexCD-oprJ*, *mexEF-oprN*, *mexGHI-opmD*, *mexJK-opmH*, *mexMN-oprM*, and *mexPQ-opmE*. Additionally, the presence of key RND pump regulator genes were found and included repressor *nalC-nalD* (for *mexAB-oprM*), repressor of efflux-complex *nfxB* (for m*exCD-oprJ*), suppressor-of-*mexT mexS* (for *mexEF-oprN*), sensor-protein *soxR* (for *mexGHI-opmD*), and repressor-of-*mexJK mexL* (for *mexJK-ompH*).

Exclusively to the mobile phone cohort, the plasmid-or-transposon-encoded-chloramphenicol-exporter *cm*x [present in 5/35 (14%) mobile phones], MFS efflux system qac MDR efflux pump qacA [present in 2/35 (6%) mobile phones] *mefA* and *mefB* (both conferring macrolide resistance) [present in 1/35 (3%) and 3/35 (9%) mobile phones, respectively], and *smeA* and *smeB* [present in 1/35 (3%) mobile phones] that compose the RND efflux pump smeABC were found. Although the *smeC* gene was not retrieved from the metagenomics, the protein kinase senso regulator *smeS* for smeABC was found in one mobile phone. Finally, *mepR*, which is a gene encoding for an upstream repressor of *mepA* in *S. aureus*, was also found in 1 mobile phone (1/35; 3%).

**Figure 6:**
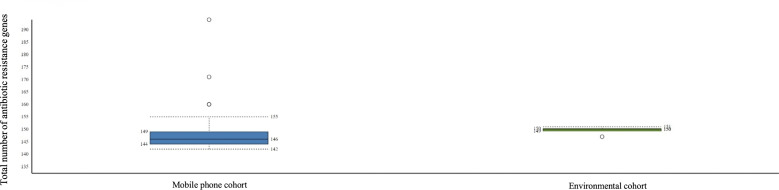
Boxplot representing the number of antibiotic-resistant genes found per sample (left: mobile phone cohort; right: environmental cohort).

Regarding aminoglycoside resistome, 11 and 3 different aminoglycoside resistance genes were found in the mobile phone cohort and the environmental cohort, respectively. In both cohorts, all swabs harbored *aph3*’ *Iib*, which encodes for an aminoglycoside phosphotransferase antibiotic-modifying enzyme. Other modifying enzyme genes such as those for adenyl transferase and acetyl transferase were mostly present in the mobile phone cohort. Regarding beta lactam resistome, 12 and 7 different beta-lactam resistance genes were found in the mobile phone cohort and environmental cohort, respectively. Beta-lactam-resistance *bla*_OXA-50_ and *bla*_PDC-2_ genes were found in all mobile phones and environmental samples. Three beta-lactam genes *CfxA* (conferring resistance to third generation cephalosporin) were found with 21 hits in the mobile phone cohort but these were absent in the environmental cohort. Additionally, beta-lactam-resistance *bla*_CARB_ (carbenicillinase) and *bla*_MOX_ (Class C AmpC-type beta-lactamase) were found with 1 hit each in the mobile phone cohort but were not identified in the environmental cohort. Four other *pdc* genes (class C beta lactamases) were found in the mobile phone cohort and environmental cohort with 43 hits and 7 hits, respectively. Beta-lactam-resistance *bla*_PAO_ gene was found in the mobile phone cohort and environmental cohort with 4 hits and 1 hit, respectively. Boxplot representation of the number of antibiotic resistance genes found between both cohorts is provided in **Figure 6**.

Data on the virulence factor genes identified in the mobile phone cohort and environmental cohort are shown in **Supplementary Table 3**.

### SARS-CoV-2 Contamination of Mobile Phones and Environmental Samples

All five mobile phones swabbed in the COVID-19 zone and 15 randomly selected mobile phones from the general zone were tested for SARS-CoV-2 contamination. One mobile phone (*n*/*N* = 1/5; 20%) from the COVID-19 zone showed positive SARS-CoV-2 detection, while all those from the general zone were SARS-CoV-2 negative. The HCW whose mobile phone showed SARS-CoV-2 contamination reported cleaning the phone at least once in the preceding 24 h with alcohol-based swab. Eight environmental samples (COVID-19 zone: *n* = 2; general zone: *n* = 6) were screened and all were SARS-CoV-2 negative.

## Discussion

Mobile phones have been described as fomites for the propagation and dissemination of microbes. Indeed, several studies using culture-based methods have described contamination of mobile phones in healthcare and community settings (Selim and Abaza, 2015; Amanah et al., 2019; Jansen et al., 2019; Khashei et al., 2019; D’Alo et al., 2020; Hikmah and Anuar, 2020; Olsen et al., 2020). In this study, our findings from culture-based methods demonstrate a high prevalence of contamination with *Staphylococcus* spp. which is in keeping with reports from other studies (Selim and Abaza, 2015; Amanah et al., 2019; Jansen et al., 2019; Khashei et al., 2019; D’Alo et al., 2020; Hikmah and Anuar, 2020). This study was carried out during the COVID-19 pandemic when heightened awareness of hand hygiene and environmental cleaning protocols was the norm. The use of the BHI, which supports growth of a wide variety of organisms, including fastidious ones in our culture-based assay was crucial to ensure detection of low biomass of microbial contaminants, which might occur due to the prevailing circumstances. Despite the adoption of this approach, our findings remained in keeping with reported literature studies (Selim and Abaza, 2015; Amanah et al., 2019; Jansen et al., 2019; Khashei et al., 2019; D’Alo et al., 2020; Hikmah and Anuar, 2020). As *Staphylococcus* spp. predominate in the skin microbiome, their transference to mobile phones is highly likely and this will explain their ubiquitous presence as contaminants. However, a major concern is the detection of multidrug-resistant staphylococcal strains and other opportunistic pathogens including pathogenic fungi on the mobile phones of HCWs. MRSA, methicillin-resistant *S. epidermidis*, and carbapenemase-producing pan-drug-resistant *A. baumannii* are frequently associated with the healthcare settings and are important agents of nosocomial infections (Carbon, 2000; Ayoub Moubareck et al., 2021). Indeed, studies from the Arabian Gulf region have shown high prevalence of MRSA colonization of nasal nares of HCWs (Iyer et al., 2014). The presence of these drug-resistant pathogens on mobile phones of HCWs poses significant risk of transmission to the patient and highlights the need for stringent infection prevention measures. The MRSA strains detected on mobile phones of HCWs in this study all belong to the same clonal complex (CC1), which is known to be prevalent in the Arabian Gulf region (Senok et al., 2016; Boswihi et al., 2018; Senok et al., 2020). A significant concern, however, is that they all harbored the fusidic acid resistance gene *fusC* on the SCC*mec* element as well as various virulence genes including adhesins and biofilm-associated genes. The SCC*mec*-associated *fusC* gene has been linked with the evolution of MRSA strains in the Arabian Gulf region, and their dissemination *via* contaminated mobile phones represents a threat to the fight against antimicrobial resistance (Senok et al., 2016; Boswihi et al., 2018; Senok et al., 2020). Furthermore, as the HCWs represent a bridge between the hospital and the community, they could carry these multidrug-resistant pathogens back to their respective communities *via* contaminated mobile phones.

In contrast to the findings from the culture-based approach where we identified mostly Gram-positive bacteria, the shotgun metagenomics analysis revealed a high signature of Gram-negative bacteria as well as presence of bacteriophages, fungi, and protists. Although previous studies on microbial contamination of mobile phones have largely used culture-based methods, Simmonds et al. (2020) recently utilized 16s rRNA sequencing approach and found a high abundance of Gram-negative organisms, which were undetected using conventional culture methods. In particular, *Acinetobacter* spp. and *Pseudomonas* spp., which were rarely detected by culture, were found at similar abundance to *Staphylococcus* spp. with *Pseudomonas* being the most abundant genus contaminating hospital phones. The findings in this current study are in keeping with this report as a significant signature of *P. aeruginosa* was found to be present on the mobile phones of the HCWs. Furthermore, it appears that trafficking between environmental surfaces and the phones might be ongoing possibly *via* hands of HCWs as similar high signature of *P. aeruginosa* was found on environmental samples and mobile phones. Our study represents the first report of shotgun metagenomics analysis of swab samples from mobile phones in which the bacterial culture step was bypassed, which therefore represents a direct mobile phone swab to metagenomic shotgun analyses. In a recent pilot study by Olsen et al. (2021b) to assess microbial contamination of mobile phones in the community, an initial bacterial culture followed by downstream DNA metagenomic next-generation sequencing of mobile phone samples from the cultures was adopted. Although the findings revealed the presence of a significant burden of viable microbes from culture, the metagenomics analysis showed predominance of Gram-positive organism in particular *Staphylococcus* spp. This contrasts with the findings in the current study, and we hypothesize that this reflects the selection for Gram-positive microbes from the bacterial culture step, which preceded the sequencing.

In addition to the detection of viable culturable microbes, the sequencing approach also includes the signature of dead as well as viable but non-culturable microbes, and this could be deemed as a limitation. However, our findings demonstrate the advantages of the shotgun metagenomics analysis as it provides a more robust dataset compared to 16s rRNA sequencing. The culture-based approach identifies only viable culturable bacteria while shotgun metagenomics identifies the whole microbial community without discriminating between viable and dead cells. However, although each of these two methods has its limitation, our findings suggest that their combined usage is desirable to elucidate important information for better understanding of microbial contamination of these digital devices. The potential capability of *Pseudomonas* spp. to enter a viable but nonculturable state has been reported for *P. putida* (Pazos-Rojas et al., 2019). We hypothesize that occurrence of similar phenomenon in *Pseudomonas* species on mobile phones could explain the observed low detection on conventional culture compared to the high signature burden observed on sequencing in this study.

The detection of multiple antimicrobial-resistant genes and virulence factor genes suggests that these microbial contaminants, in particular the predominant *P. aeruginosa* strains, are potentially highly pathogenic. Majority of the viruses detected were bacteriophages, and their presence raises the possibility of horizontal gene transfer to unrelated bacteria. The presence of antibiotic-resistant genes especially those for efflux multidrug efflux pump systems and genetic transfer-mediating genes is really of significant concern. This potential for horizontal gene transfer provides support for the recommendation that institution of policies on the usage of mobile phones in the workplace by HCWs and infection prevention protocols for adequate cleaning of these devices are warranted. We envisage that the findings from this study will be a stimulus for raising awareness towards the development of such policies and protocols in our setting. However, further studies to investigate occurrence of horizontal gene transfer mechanisms and bacterial biofilm formation on the surfaces of mobile phones are urgently needed.

In contrast to other reported work, the environmental swabs in this study were negative for SARS-CoV-2 contamination in both the general and COVID-19 zones (Santarpia et al., 2020). This finding is significant as COVID-19 patients who seek medical attention in the emergency unit are more likely to be symptomatic with high viral load and higher risk of viral shedding into the environment. While the identification of a single contaminated phone in the COVID-19 zone raises concern as it confirms that mobile phones are platforms capable of harboring SARS-CoV-2, it should also be recognized that the viral viability was not determined in this study. Nevertheless, the findings suggest that there might be ease of acquisition and retention of SARS-CoV-2 contamination on mobile phones in hospital areas where COVID-19 patients are being cared for. Although environmental decontamination appears to be effective as we did not detect any SARS-CoV-2 on the environmental samples and mobile phones, stringent measures regulating use and disinfection of mobile phones particularly for HCWs in working COVID-19 high risk areas is recommended. In this study, we did not take samples from the air as airflow changes across the emergency unit; hence, such sampling is unlikely to be representative. The surfaces are the sites where mobile phones are more likely to get contaminated due to frequent contact; hence, samples from multiple surfaces across the unit provide a comprehensive snapshot of the contaminants present.

In conclusion, our findings demonstrate that mobile phones of HCWs in a busy emergency unit of a tertiary care facility are fomites for potentially pathogenic and highly drug-resistant microbes. Mobile phones are contaminated platforms that negate handwashing practices with the potential for transference to patients and the general community representing a significant public health risk. The introduction of highly efficient phone sanitization methods, such as ultraviolet C sanitization devices, in hospitals and public areas should be investigated as approaches for mitigatation against the risk of microbial mobile phone contamination and dissemination.

## Data Availability Statement

The datasets presented in this study can be found in online repositories. The names of the repository/repositories and accession number(s) can be found at: https://www.ncbi.nlm.nih.gov/genbank/; BioProject ID: PRJNA750471.

## Ethics Statement

The studies involving human participants were reviewed and approved by Dubai Scientific Research Ethics Committee, Dubai Health Authority (REF#: DSREC-02/2021_02) MBRU Institutional Review Board (REF#: MBRU-IRB-2020-040). Written informed consent for participation was not required for this study in accordance with the national legislation and the institutional requirements.

## Author Contributions

RN, AS, RA, and LT: Conceptualization and study design, data analysis and interpretation, drafting and critical revision of the manuscript. MO, MC, PJ, and SM: Data analysis and interpretation, critical revision of manuscript. SB, SZ, FA, LM, MH, and AA: Data collection, collation and interpretation, critical revision of manuscript. All authors contributed to the article and approved the submitted version.

## Funding

This study is funded by internal research grant from Mohammed Bin Rashid University of Medicine and Health Sciences (Ref#:MBRU-CM-RG2019-03).

## Conflict of Interest

The authors declare that the research was conducted in the absence of any commercial or financial relationships that could be construed as a potential conflict of interest.

## Publisher’s Note

All claims expressed in this article are solely those of the authors and do not necessarily represent those of their affiliated organizations, or those of the publisher, the editors and the reviewers. Any product that may be evaluated in this article, or claim that may be made by its manufacturer, is not guaranteed or endorsed by the publisher.
