# Supplementary material for: Metagenomic Sequencing and Reverse Transcriptase PCR Reveal That Mobile Phones and Environmental Surfaces Are Reservoirs of Multidrug-Resistant Superbugs and SARS-CoV-2
Source: Front Cell Infect Microbiol. 2022 Mar 8;12:806077. doi: 10.3389/fcimb.2022.806077 (PMC8964345; doi:10.3389/fcimb.2022.806077)
Supplement: Supplementary file 1 [file DataSheet_1.pdf]

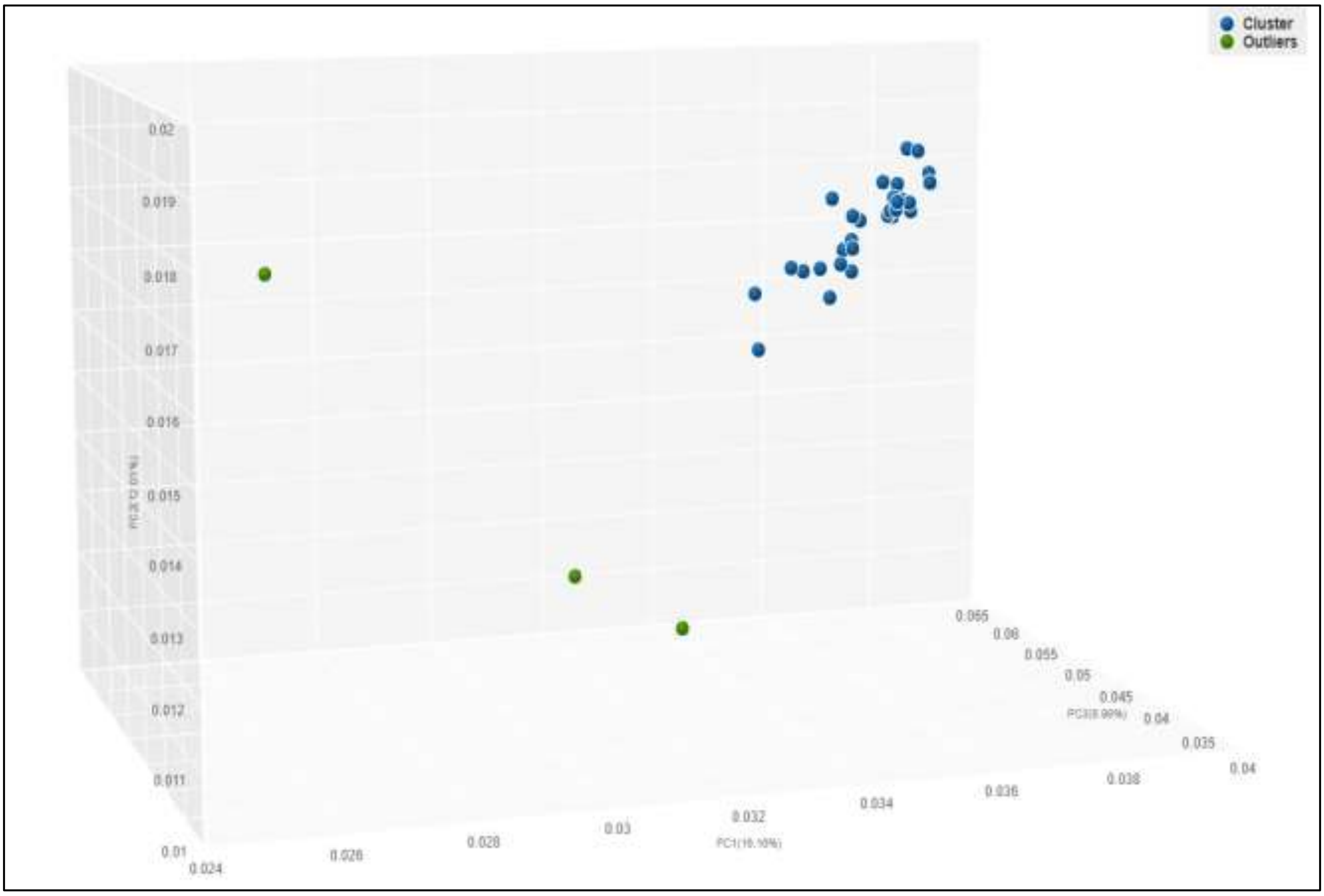

Supplementary Figure: Principal component analysis (PCA)

A large cluster is found among all mobile phone samples with the exception of three outliers shown in dots in green
