# Supplementary material for: Metagenomic Sequencing and Reverse Transcriptase PCR Reveal That Mobile Phones and Environmental Surfaces Are Reservoirs of Multidrug-Resistant Superbugs and SARS-CoV-2
Source: Front Cell Infect Microbiol. 2022 Mar 8;12:806077. doi: 10.3389/fcimb.2022.806077 (PMC8964345; doi:10.3389/fcimb.2022.806077)
Supplement: Supplementary file 2 [file DataSheet_2.pdf]

Supplementary Table 1: Molecular characterization of *Staphylococcus aureus* strains

Clonal complex	Strain assignment	SCC *mec*-complex-associated genes	Regulatory & capsule genes	Antibiotic resistance genes	Toxin associated virulence genes	Other virulence genes
**CC1 (n=3)**						
	CC1-MRSA-[V+*fusC*+*ccrAB1*] (n=2)	*ugpQ; mecA; ccrA-1; ccrB-1; ccrAA; Q6GD50 (fusC)*	*agrIII; cap8*	*aacA-aphD*	*sea; seb; seh; sek; seq*	*hla; sak; scn; icaA/C/D; clfA/B; fnbA/B; cna; sasG*
	CC1-MRSA-[V/VT+fus+ccrAB1] (n=1)	*ugpQ; mecA; Q6GD50 (fusC); ccrA-1; ccrB-1;*	*agrIII; cap8*	*aacA-aphD*	*sea; seb; seh;*	*hla; sak; scn; icaA/C/D; clfA/B; fnbA/B; cna; sasG*
**CC15 (n=1)**	CC-15 MSSA	--	*agrII; cap5*	*blaZ; blaI; blaR; fosB*	-	*hla; chp; scn; etA; icaA/C/D; clfA/B; fnbA/B; sasG*
**CC361 (n=1)**	CC-361 MSSA	--	*agrI; cap8*	*fosB*	*egc cluster*	*hla; sak; chp; scn; icaA/C/D; clfA/B; fnbA/B; sasG*

agr, accessory gene regulator*; ccr,* cassette chromosome recombinase gene*; blaZ*, beta-lactamase; *blaI*, beta lactamase repressor (inhibitor); *blaR,* beta-lactamase regulatory protein*; can*, collagen binding adhesin *etA,* exfoliative toxin A, *sea,* enterotoxin A*; seb,* enterotoxin B, *sed,* enterotoxin H, *sek,* enterotoxin K*, seq,* enterotoxin Q*, egc cluster:* enterotoxins g,i,m,n,o,u; *sak,* staphylokinase*; scn,* staphylococcal complement inhibitor*; chp,* chemotaxis-inhibiting protein (CHIPS)*; hla*, haemolysin alpha*; cna,* collagen-binding adhesin*; fnbA,* fibronectin-binding protein A*; fnbB,* fibronectin-binding protein B*; icaA/C/D*, intercellular adhesion protein; *sasG*, *Staphylococcus aureus* surface protein G; *mecA,* alternate penicillin binding protein 2, defining MRSA*; Q6GD50 (fusC),* hypothetical protein associated with fusidic acid resistance *aacA-aphD,* bifunctional enzyme for gentamicin resistance; *fosB*, Metallothiol transferase
