# Supplementary material for: Metagenomic Sequencing and Reverse Transcriptase PCR Reveal That Mobile Phones and Environmental Surfaces Are Reservoirs of Multidrug-Resistant Superbugs and SARS-CoV-2
Source: Front Cell Infect Microbiol. 2022 Mar 8;12:806077. doi: 10.3389/fcimb.2022.806077 (PMC8964345; doi:10.3389/fcimb.2022.806077)
Supplement: Supplementary file 3 [file DataSheet_3.pdf]

Supplementary Table 2: Bacteria dectected in the three outliers found in the principal component analysis of the mobile phone cohort

Hits	Name	Hits	Name
100% (3/3)	*Acinetobacter_u_t*	100% (3/3)	
100% (3/3)	*Actinomyces naeslundii str. Howell 279*	100% (3/3)	*Micrococcus sp. RIT608*
100% (3/3)	*Actinomyces oris K20*	100% (3/3)	*Micrococcus yunnanensis*
100% (3/3)	*Actinomyces viscosus C505*	100% (3/3)	*Moraxella osloensis*
100% (3/3)	*Agrobacterium tumefaciens F2*	100% (3/3)	*Paenibacillus sophorae S27*
100% (3/3)	*Aquabacterium parvum*	100% (3/3)	*Porphyromonadaceae bacterium KA00676*
100% (3/3)	*Corynebacterium pseudogenitalium ATCC 33035*	100% (3/3)	*Porphyromonas catoniae*
100% (3/3)	*Corynebacterium tuberculostearicum SK141*	100% (3/3)	*Prevotella_u_t*
100% (3/3)	*Enhydrobacter aerosaccus SK60*	100% (3/3)	*Pseudomonas aeruginosa 6077*
100% (3/3)	*Enhydrobacter sp. H5*	100% (3/3)	*Pseudomonas sp. HMSC063H08*
100% (3/3)	*Haematobacter massiliensis*	100% (3/3)	*Pseudomonas_u_t*
100% (3/3)	*Haemophilus parainfluenzae ATCC 33392*	100% (3/3)	*Pseudopropionibacterium propionicum F0230a*
100% (3/3)	*Haemophilus_u_t*	100% (3/3)	*Rothia dentocariosa M567*
100% (3/3)	*Lautropia mirabilis ATCC 51599*	100% (3/3)	*Schaalia odontolytica ATCC 17982*
100% (3/3)	*Micrococcaceae bacterium JKS001869*	100% (3/3)	*Streptococcus_u_t*
100% (3/3)	*Micrococcus aloeverae*	66.6% (2/3)	*Stenotrophomonas sp. MB339*
