# Supplementary material for: Metagenomic Sequencing and Reverse Transcriptase PCR Reveal That Mobile Phones and Environmental Surfaces Are Reservoirs of Multidrug-Resistant Superbugs and SARS-CoV-2
Source: Front Cell Infect Microbiol. 2022 Mar 8;12:806077. doi: 10.3389/fcimb.2022.806077 (PMC8964345; doi:10.3389/fcimb.2022.806077)
Supplement: Supplementary file 4 [file DataSheet_4.pdf]

Detection of Virulence factor genes (VFGs):

A total of 251 and 164 different VFG genes were found in mobile phone cohort and environmental cohort respectively. The number of VFGs omnipresent in every single swab sample was 142 in the mobile phone cohort and 144 in the environmental cohort. All VFGs which were present in the mobile phone cohort were also found in the environmental cohort. However, 7 VFGs were exclusively found in the environmental cohort (i.e. absent in mobile phone cohort) and these are *korC* (*Enterobacter aerogenes*), *tniB* (*E. aerogenes*), *tetL* (*Enterococcus faecalis*), *ica* (*S. aureus*), and *repL* (*Staphylococcus lentus*).

There was a high predominance of VFGs *quasi*-exclusively associated with *P. aeruginosa* (mobile phone cohort: 142/142 VFGs; environmental cohort: 143/144 VFGs). Of note, in the environmental cohort the remaining VGF was from *Salmonella Typhimurium*. *P. aeruginosa* related VFGs included a diverse range of functional mechanisms with 27 motility related genes, 31 adherence and aggregation related genes, 29 biofilm related genes, 19 toxin/pigment biosynthesis related genes, 17 secretion machinery systems related genes and 8 secreted proteins related genes as shown in Supplementary Table 3.

Supplementary Table 3: Pseudomonas virulent factors genes found in mobile phones and environmental swabs

**Motility**	**Adherence & Aggregation**	**Biofilm/ Quorum sensing/ Genetic Transfer**
fleN, fleQ, fleR	*chpB, chpC, chpD, chpE*	*Alg4, Alg8, AlgA, AlgB, AlgD, AlgE, AlgF, AlgG, AlgI*
flgC, flgD, flgE, flgF, flgG, flgH,flgI, flgJ	*fimT, fimU, fimV.*	*AlgJ, AlgK, AlgL, AlgP, AlgQ, AlgR, AlgU, AlgX, AlgZ*
flhA, flhB, flhF	*pilD, pilE, pilF, pilD, pilE, pilF, pilG, pilH, pilI, pilJ, pilM, pilN*	*mucA, mucB, mucC*
fliE, fliF, fliG, fliH, fliI, fliJ, fliM, fliN, fliE, fliO, fliP, fliQ, fliR.	*pilO, pilP, pilQ, pilR, pilS, pilT, pilU, pilV, pilW, pilX, pilY2, pilZ.*	*lasI, lasR, rhlL, rhlR, orfA, tnpA, tnpR, intl1*
**Toxin/pigment biosynthesis**	**Secretory System**	**Secreted compound to manipulate Host Cells or/and for**

		**cytotoxicity/tissue damage**
waaA, waaC, waaF, waaG, waaP	*T2SS: xcpP, xcpQ, xcpR, xcpS, xcpT, xcpU, xcpV, xcpW, xcpY, xcpZ*	*toxA, plcH lasA, lasB (secreted by T2SS), aprA (secreted by T1SS)*
fptA	*T3SS: ExsC*	*exoT and exoY, (secreted by T3SS)*
pchA, pchB, pchC, pchD, pchE, pchF, pchG, pchH, pchI, pchR	*T6SS: vgrG1, icmF1, ppkA, clpV1,*	*Hcp1 (secreted by T6SS)*
pvdS, phzM, phzS	*Surfactant: rhlA, rhlB*	
